# Paxillin and Kindlin: Research Progress and Biological Functions

**DOI:** 10.3390/biom15020173

**Published:** 2025-01-24

**Authors:** Zijian Li, Ruonan Shao, Honglei Xin, Yilin Zhu, Suyu Jiang, Jiao Wu, Han Yan, Tongyu Jia, Mengyu Ge, Xiaofeng Shi

**Affiliations:** The Second Affiliated Hospital of Nanjing Medical University, No.262, North Zhongshan Road, Nanjing 210003, China; hxfysmm@stu.njmu.edu.cn (Z.L.);

**Keywords:** paxillin, kindlin, cell adhesion, integrin activation, focal adhesion, tumor metastasis, immune response, therapeutic targets

## Abstract

Paxillin and kindlin are essential regulatory proteins involved in cell adhesion, migration, and signal transduction. Paxillin influences cytoskeletal dynamics by interacting with multiple signaling proteins, while kindlin regulates integrin activation, affecting adhesion and motility. This review examines the structures and functions of these proteins, focusing on their roles in cancer progression, immune response, and therapeutic potential. The cooperation between paxillin and kindlin in integrin activation and focal adhesion dynamics offers valuable insights into tumor metastasis, immune function, and tissue repair.

## 1. Introduction

Signal transduction is a critical biological mechanism that governs cell adhesion, migration, and tissue morphogenesis. Focal adhesions (FAs) serve as structural and signaling hubs, connecting cells to the extracellular matrix (ECM) and enabling dynamic cellular interactions.

Recent research has highlighted the interaction between paxillin and kindlin as essential for cell adhesion and migration. Paxillin regulates dynamic changes in the cytoskeleton through its binding to various signaling proteins, whereas kindlin influences cell adhesion and motility by modulating integrin activation. Therefore, understanding the coordinated roles of paxillin and kindlin is pivotal for deciphering the molecular mechanisms underlying cell adhesion and migration.

This review aims to examine the relationship between paxillin and kindlin and their specific roles in cellular signal transduction. It explores their functions under both normal physiological and pathological conditions, with a special emphasis on their involvement in tumor initiation, progression, and the immune system. Additionally, we provide an overview of recent research advances in this field.

## 2. Structure and Function of Paxillin

### 2.1. Molecular Structure of Paxillin

Paxillin is a protein composed of 559 amino acids and has a molecular weight of 68 kDa. It serves as a multifunctional adaptor protein at FAs, primarily regulating cytoskeletal reorganization, cell migration, and signal transduction. The structure of paxillin includes specific domains, such as LD and LIM motifs, which enable interactions with signaling molecules and FA proteins, supporting its regulatory roles in cellular processes [1].

The N-terminal region of paxillin contains five conserved LD motifs (LD1–LD5), which mediate interactions with FA proteins such as talin and vinculin, supporting adhesion assembly. The C-terminal LIM domains are zinc-finger-like structures that facilitate nuclear localization and cytoskeletal signaling [2].

**Figure 1:**
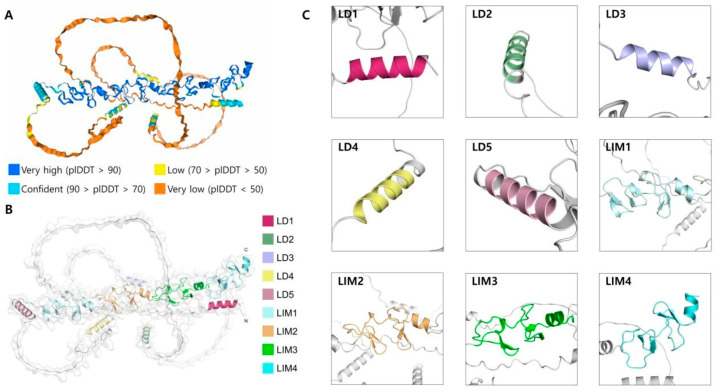
Modeling and display of paxillin protein structure based on AlphaFold Server (https://alphafoldserver.com, accessed on 3 November 2024). (**A**) The three-dimensional structure of paxillin protein generated by AlphaFold3, along with the per-residue confidence score (pLDDT), where higher values indicate greater confidence. The predicted template modeling score (pTM) for this model is 0.39. (**B**) The major structural domains and motifs of paxillin based on the sequence annotations from UniProt (paxillin_human), with the UniProt sequence ID P49023. (**C**) The structural representation of paxillin’s LD1-5 and LIM1-4 domains.

The C-terminal region contains four LIM domains (LIM1-LIM4), zinc finger-like structures involved in protein–protein interactions. These domains are not only localized at FA sites but also play a role in paxillin’s nuclear localization. For instance, LIM domains can associate with nuclear proteins, suggesting a potential role for paxillin in transcriptional regulation [3,4]. For instance, LIM domains can interact with nuclear proteins, suggesting a role for paxillin in transcriptional regulation. Additionally, these domains facilitate protein–protein interactions that modulate cytoskeletal dynamics and signal transduction [5,6]. Figure 1 shows the three-dimensional structural model of paxillin, and its individual domains predicted through computational modeling by AlphaFold3.

### 2.2. The Biological Functions of Paxillin

#### 2.2.1. Regulation of Integrins

Integrins, as transmembrane receptors, are central in mediating cellular interactions with the ECM, critical for signal transduction, cytoskeletal dynamics, and motility. Through their intracellular tails, integrins engage FA proteins like paxillin, mediating both “inside-out” and “outside-in” signaling and regulating mechanical response and signal transduction [7,8]. Upon ECM adhesion, integrins activate, triggering downstream signaling that drives FA assembly and maturation [9,10]. The C-terminal LIM domain of paxillin binds directly to integrin β subunits, enhancing adhesion complex stability and fortifying cell–ECM connections [6,11]. By modulating integrin activation, paxillin supports early-adhesion site formation and stabilizes cytoskeletal reorganization via downstream pathways.

#### 2.2.2. Focal Adhesion Assembly and Disassembly

Paxillin plays a central role in both the assembly and disassembly of FAs. During adhesion formation, the N-terminal LD motif of paxillin binds to talin and vinculin, stabilizing adhesion structures and connecting the cytoskeleton to the ECM [12,13]. Paxillin also promotes actin reorganization by regulating members of the Rho family GTPases, including RhoA, Rac1, and Cdc42, which are key regulators of cytoskeletal dynamics [14,15]. In the disassembly phase, Src family kinases (SFKs) phosphorylate paxillin, which initiates a phosphorylation cascade to facilitate cell migration. This balance between assembly and disassembly, regulated by paxillin, is essential for efficient cell motility [16].

#### 2.2.3. Paxillin Is Involved in Regulating Signal Transduction

Paxillin interacts with the Rho family of GTPases (such as RhoA, Rac1, and Cdc42) indirectly by modulating upstream regulators, specifically guanine nucleotide exchange factors (GEFs) and GTPase-activating proteins (GAPs). As a scaffold protein located at FAs, paxillin integrates ECM signals with intracellular pathways. When phosphorylated, paxillin recruits various signaling molecules to FA sites, spatially and temporally regulating downstream signaling. Paxillin, through its interactions with GEFs, promotes the activation of Rho GTPases by facilitating the exchange of GDP to GTP, leading to cytoskeletal reorganization and enhancing cell motility [17]. Conversely, paxillin also interacts with GAPs, which inactivate Rho GTPases by enhancing their intrinsic GTPase activity, converting GTP to GDP. This dual regulation ensures precise control over cytoskeletal dynamics, including stress fiber formation via RhoA, lamellipodia formation via Rac1, and filopodia formation via Cdc42. The phosphorylation state of paxillin, particularly at key tyrosine residues (e.g., Y31 and Y118), plays a critical role in determining its interactions with GEFs and GAPs, thereby modulating the activation states of Rho GTPases. While paxillin does not directly bind to Rho GTPases, it influences their activity indirectly by acting on their regulatory proteins. This interplay creates a dynamic feedback loop where active Rho GTPases regulate kinases like FAK and Src, which in turn phosphorylate paxillin, facilitating a coordinated response essential for cell adhesion, migration, and cytoskeletal reorganization [18].

Paxillin plays a critical role in regulating FA turnover by orchestrating the recruitment and activity of specific proteins involved in both the assembly and disassembly of FAs. During FA assembly, paxillin recruits key signaling molecules such as focal adhesion kinase (FAK) and Src, which initiate FA formation. Upon phosphorylation at tyrosine residues like Y31 and Y118, paxillin acts as a docking site for proteins involved in actin polymerization and integrin clustering, stabilizing nascent adhesions and promoting their maturation. Additionally, paxillin facilitates the recruitment of structural proteins such as vinculin and talin, which link integrins to the actin cytoskeleton and ensure structural integrity during adhesion formation. In contrast, paxillin also plays a crucial role in FA disassembly, where it facilitates detachment by regulating the activity of proteins such as calpain, which cleaves adhesion components, and p190RhoGAP, which suppresses RhoA activity [19]. This suppression reduces actomyosin contractility, promoting adhesion disassembly. Furthermore, the dephosphorylation of paxillin by phosphatases such as PTP-PEST destabilizes FAs and triggers their turnover. The dual role of paxillin in assembly and disassembly is tightly regulated by its phosphorylation status and interactions with signaling and structural proteins. During assembly, paxillin stabilizes and recruits essential components to the adhesion site, while in disassembly, it destabilizes adhesions by modulating proteolysis, cytoskeletal tension, and integrin detachment, ensuring dynamic remodeling necessary for cell migration [17,20,21].

#### 2.2.4. Paxillin Functions in Regulating Cell Migration

Paxillin plays a crucial role in cell migration by regulating both the assembly and disassembly of FAs, processes essential for initiating, maintaining, and completing cell migration.

At the leading edge of migrating cells, paxillin facilitates the assembly of FAs, providing the necessary adhesion force to propel the cell forward. This function is supported by its interactions with various signaling molecules, including FAK and SFKs, which are recognized for their ability to regulate actin cytoskeletal dynamics. For example, the phosphorylation of paxillin at particular residues, such as Ser273, has been demonstrated to strengthen its interaction with other proteins involved in adhesion dynamics, thereby promoting cell migration [22,23]. Additionally, the mechanosensitive properties of paxillin allow it to respond to mechanical cues from the ECM, further influencing the direction and velocity of cell migration [24].

Conversely, at the trailing edge of migrating cells, paxillin is pivotal in facilitating the disassembly of FAs. The phosphorylation state of paxillin is crucial in mediating this process, allowing cells to release their connections with the ECM and complete the migration cycle. For example, studies have demonstrated that paxillin phosphorylation controls adhesion turnover, a process critical for efficient cell movement [22]. Furthermore, the interplay between paxillin and Rho family GTPases, including RhoA, Cdc42, and Rac1, is essential for coordinating the cytoskeletal reorganization necessary for migration [25]. Because they regulate various aspects of actin dynamics, including the formation of stress fibers, the extension of lamellipodia, and filopodium formation [26], the activation of these GTPases is stringently controlled by GEFs and GAPs, which modulate their activity in response to external signals [27]. The interplay between paxillin and these regulatory proteins ensures that cells can effectively respond to environmental changes, facilitating efficient migration through complex tissue architectures [28].

Paxillin also functions as a mechanosensor. Through its LIM domains, it senses and responds to external mechanical forces, further regulating the direction and speed of cell migration. This role is particularly important in tumor cell invasion and metastasis, as it enhances the migratory capacity of tumor cells by enabling their adaptive responses to mechanical forces [29,30].

### 2.3. Regulatory Mechanism of Paxillin

Paxillin’s function is tightly regulated by phosphorylation and dephosphorylation, which influence its activity and binding affinity with other proteins. These modifications are essential for modulating cell adhesion and migration. The phosphorylation of paxillin is mediated primarily by several key kinases, the most important of which are FAK and SFKs.

#### 2.3.1. Regulation of Paxillin Phosphorylation by FAK and Src

FAK, a central kinase at FA sites, phosphorylates paxillin at tyrosine residues Tyr31 and Tyr118, which enhances its binding to other signaling molecules and regulates cytoskeletal reorganization. The activation of FAK not only promotes the role of paxillin in cytoskeletal reorganization but also transmits integrin signals to downstream signaling pathways via paxillin. The dynamic interplay between FAK and paxillin promotes the recycling and translocation of FA proteins, thereby influencing cell motility and adhesion [31,32,33].

Members of the Src family of kinases (such as Src and Yes) serve as crucial modulators of paxillin. Src kinases can further phosphorylate tyrosine sites on paxillin, increasing the interaction of paxillin with FAK, vinculin, and other FA proteins. This process is essential for both the formation and disassembly of FA sites [34,35]. The activity of SFKs is regulated by extracellular signals, such as mechanical forces or changes in the ECM, suggesting that the function of paxillin depends not only on intracellular signaling networks but also on the extracellular microenvironment [36].

#### 2.3.2. Regulation of Paxillin by Serine/Threonine Kinases

Serine/threonine kinases, such as PKC, also regulate paxillin phosphorylation. Protein kinase C (PKC) is capable of phosphorylating specific serine residues of paxillin, thereby regulating its role in cell migration [37]. The PKC-mediated phosphorylation of paxillin promotes FA disassembly, a crucial step for cell migration by facilitating detachment from the ECM, thus promoting the migration process. Studies have shown that specific phosphorylation sites on paxillin, including Ser178 and Ser85, are essential for modulating its interactions with other signaling molecules, thereby regulating cellular migration. For example, the Ser178 site of paxillin is phosphorylated by JUN N-terminal kinases, and this phosphorylation event is considered vital for both cell migration and neurite outgrowth [38,39].

Kinases in the ERK/MAPK pathway phosphorylate paxillin, translating signals from external growth factors or cellular stress into adhesion complex responses. For example, studies have shown that extracellular signal-regulated kinase (ERK) activation leads to increased phosphorylation of paxillin, which enhances its association with FAK, thereby promoting cell adhesion and migration [40,41,42]. Paxillin phosphorylation facilitates its pivotal functions in cell proliferation, survival, and response to external stress signals.

### 2.4. Functions of Paxillin in Different Cell Types

Paxillin plays a pivotal role in regulating cellular adhesion and signaling across various cell types, including epitheliumfibroblasts, immunocyte, and tumor cells, with unique mechanisms tailored to each context. In epithelial cells, paxillin contributes significantly to the regulation of cell–cell junctions, particularly adherens junctions and tight junctions, which are crucial for maintaining epithelial integrity and barrier function. While its role in FAs has been extensively studied, the involvement of paxillin in cell–cell junction dynamics remains less explored but equally important. Paxillin interacts with key components of adherens junctions, such as E-cadherin and β-catenin, facilitating the stabilization and remodeling of these junctions during epithelial morphogenesis and wound healing.

Moreover, paxillin integrates mechanical cues from the extracellular environment into intracellular signaling pathways, influencing cytoskeletal reorganization and epithelial cell polarity. For example, paxillin-mediated regulation of Rho GTPases, particularly Rac1 and RhoA, is critical for balancing the forces required for junctional stability and plasticity. Additionally, paxillin’s interaction with the mitogen-activated protein kinase (MAPK) and phosphoinositide-3-kinase/activation of protein kinase B (PI3K/Akt) pathways highlights its role in coordinating cell proliferation and survival in epithelial tissues.

Despite these known roles, more in-depth research is required to elucidate paxillin’s specific contributions to epithelial cell–cell junctions under physiological and pathological conditions, such as during cancer metastasis, where epithelial-to-mesenchymal transition (EMT) disrupts junction integrity. Addressing these gaps will provide a more comprehensive understanding of paxillin’s function in epithelial biology and its potential as a therapeutic target in diseases involving epithelial dysfunction.

#### 2.4.1. Epithelial Cells

In epithelial cells, paxillin regulates both cell–cell and cell–ECM adhesion, thereby maintaining the structural stability of tissues. When epithelial cells are stimulated by mechanical forces or other external signals, paxillin undergoes rapid phosphorylation, triggering the formation of FAs and enhancing cellular attachment by regulating the interaction between integrins and vinculin [43,44]. Moreover, paxillin is vital in wound healing, especially during the accelerated phases of cell migration and proliferation. Rho GTPases, such as RhoA, Rac1, and Cdc42, are known to control various cellular processes, including actin polymerization and contractility, thereby influencing cell movement and junction stability [45,46,47]. The collaboration between paxillin and Rho GTPases accelerates the healing process by promoting the necessary cytoskeletal rearrangements that facilitate cell migration to the wound site [48].

#### 2.4.2. Immune Cells

Paxillin plays a crucial role in immunocyte function by regulating cell adhesion, migration, and signaling pathways essential for effective immune responses. It serves as a key adaptor protein at FAs, linking integrins to the actin cytoskeleton and coordinating intracellular signaling cascades required for leukocyte adhesion and movement. In immune cells, paxillin interacts with β2 integrins, which are critical for leukocyte adhesion to the vascular endothelium during immune surveillance and inflammation. Through phosphorylation, paxillin facilitates the recruitment of signaling molecules such as focal FAK and Src, which modulate cytoskeletal dynamics necessary for firm adhesion and transmigration. Additionally, paxillin regulates immunocyte migration by influencing the assembly and disassembly of FAs in a spatially and temporally-controlled manner. By interacting with Rho family GTPases such as Rac1 and RhoA, paxillin coordinates actin cytoskeleton remodeling, promoting lamellipodia formation at the leading edge and contractility at the cell rear, both essential for directed motility.

The critical role of paxillin in immunocyte trafficking is underscored in pathological conditions such as leukocyte adhesion deficiency (LAD), a genetic disorder characterized by defective integrin-mediated adhesion and the migration of leukocytes. In LAD, mutations in integrin β2 disrupt its interaction with paxillin, impairing leukocyte adhesion to the endothelium and preventing effective migration to sites of infection or inflammation. This dysfunction leads to recurrent infections and poor wound healing, highlighting the importance of paxillin in immune responses. Beyond LAD, dysregulated paxillin signaling is implicated in chronic inflammatory and autoimmune diseases, where abnormal leukocyte migration contributes to pathology. Targeting paxillin-mediated pathways offers a promising therapeutic avenue to restore proper immune cell function and address immune-related disorders. Further research on paxillin’s role in immune cells could provide insights into novel treatments for these conditions [49,50,51].

Additionally, paxillin regulates cytoskeletal rearrangements during key immune functions, such as phagocytosis. Its role in organizing the cytoskeleton allows macrophages and neutrophils to effectively engulf pathogens, demonstrating the importance of paxillin in both immune cell migration and cytoskeletal dynamics [52]. Paxillin’s function is also vital for immune cell plasticity, enabling cells such as macrophages and neutrophils to adapt to different stimuli and environments, which is essential for their role in immune defense and inflammation resolution [50,51].

#### 2.4.3. Tumor Cells

Paxillin upregulation in tumor cells is closely linked to invasiveness and metastasis. In cancers from the breast, lung, and colon, paxillin overexpression enhances tumor cell adhesion to the ECM, promoting migration and invasion [36,53,54,55]. Moreover, paxillin also enhances tumor cell proliferation and survival by regulating the PI3K/Akt and MAPK signaling pathways [56,57].

Paxillin plays a critical role in tumor metastasis by facilitating cell detachment, migration, and invasion through its interaction with FAK and Src. Phosphorylated paxillin strengthens FAs, enhancing the connection between tumor cells and ECM. This stabilizes integrin–ECM interactions, providing traction for tumor cell movement. Additionally, paxillin regulates cytoskeletal reorganization by activating Rho family GTPases, promoting the formation of lamellipodia and filopodia, structures necessary for invasive migration. These processes enable tumor cells to detach from the primary tumor site, degrade the ECM, and navigate through the stromal environment to colonize distant tissues, driving metastasis [58,59]. In addition, paxillin phosphorylation regulates other signaling molecules related to the tumor microenvironment, further enhancing the invasive capacity of the tumor [54].

## 3. The Structure and Function of Kindlin

### 3.1. The Structure of Kindlin

Kindlins are members of a family containing FERM, comprising three main subtypes: kindlin-1, kindlin-2, and kindlin-3. Each has distinct tissue-specific expression and functional roles. These subtypes share a conserved structure composed of three interconnected FERM subdomains (F1, F2, and F3), a coiled-coil region, an N-terminal F0 subdomain, and a pleckstrin homology (PH) domain embedded within the F2 subdomain [60]. In addition to these features, kindlins directly interact with actin cytoskeleton components through their K2 domain, a function highlighted in studies by Bledzka et al. [61]. This actin-binding property is crucial for their role in integrin activation and cytoskeletal dynamics [62].

The F1 subdomain contributes to the structural integrity of kindlin by working alongside other domains to maintain protein stability. Studies have shown that this region may engage in weak interactions with certain cytoplasmic proteins, although its ability to directly bind targets is relatively low [63]. The F2 subdomain contains a PH domain, which enables kindlin to interact with phospholipids, particularly polyphosphoinositides, such as phosphatidylinositol (PtdIns) [64]. This domain is crucial for the localization of kindlin to the plasma membrane. This interaction allows kindlin to respond rapidly to extracellular signals through changes in membrane signaling [65]. The F3 subdomain is a key component within the structure of kindlin, as it directly interacts with the intracellular tail of integrins [66]. Integrins are transmembrane heterodimeric receptors critical for cell adhesion and signal transduction. Kindlin’s F3 subdomain regulates integrin activation by attaching to the NPXY (Asn-Pro-Xaa-Tyr) motif of integrins, participating in “inside-out” integrin signaling [67].

### 3.2. The Functions of Kindlin

#### 3.2.1. The Roles of Different Kindlin Isoforms

Members of the kindlin family play distinct roles in specific tissues and cell types. Kindlin-1 is predominantly found in skin and epithelial cells, and mutations in this protein are linked to Kindler Syndrome (KS), a rare genetic condition characterized by skin fragility and pigmentation defects. The loss of kindlin-1 leads to adhesion dysfunction in skin cells, resulting in symptoms such as blistering and chronic ulcers [68]. Kindlin-2 is more widely expressed, including in fibroblasts, epithelial cells, and muscle cells. It is involved primarily in cytoskeletal reorganization and cell migration, and its absence results in embryonic lethality, indicating its critical role in embryonic development [69]. Additionally, research has demonstrated that kindlin-2 expression is elevated in tumor cells and plays a role in cancer cell invasion and metastasis [70,71]. Kindlin-3 is expressed in blood and immune cells, and its mutations are linked to Leukocyte Adhesion Deficiency type III (LAD III), a severe disorder affecting immune cell function [72]. Kindlin-3 is crucial for the activation of platelet integrins, which serve key functions in both hemostasis and the immune response. It regulates the adhesion and migration functions of immune cells by interacting with the integrin β subunit [73,74].

#### 3.2.2. The Role of Kindlin in Integrin Activation

Integrins facilitate cellular–ECM interactions through their intracellular tails, which bind directly to kindlin, an essential integrin coactivator. Integrin activation occurs via two distinct modes: “inside-out”, where intracellular signals activate integrins to bind ECM ligands, and “outside-in”, where ECM binding triggers intracellular signaling pathways. In these processes, kindlin not only enhances integrin affinity for ECM ligands but also coordinates the intracellular signaling cascades essential for cellular adhesion, migration, and survival.

In “inside-out” activation mode, kindlin binds directly to the β subunit of integrins through its F3 domain, increasing their affinity for ECM ligands. This process is typically initiated by external signals, such as chemokines or growth factors, which activate intracellular pathways, such as the PI3K/Akt pathway, promoting the binding of kindlin to integrins [60,75].

In “outside-in” activation, after integrins bind to the ECM, intracellular signaling pathways are activated, including the phosphorylation of FAK and SFKs, which subsequently drives cytoskeletal reorganization and promotes cell migration. In this “outside-in” signaling mode, kindlin assists in maintaining integrin activity and coordinates with other FA proteins, including talin, to modulate the assembly and disassembly of FA complexes [67,75].

Moreover, the role of kindlin is not limited to facilitating integrin activation; it also participates in the clustering of integrins, which is vital for stabilizing FAs. This clustering enhances the avidity of integrins for multivalent ligands, thereby strengthening cell adhesion [67,76]. Furthermore, the interactions of kindlin with proteins such as integrin-linked kinase (IIK) underscore its importance in integrin-mediated signaling and cellular mechanics [77].

#### 3.2.3. Role of Kindlin in Cytoskeleton Remodeling and Migration

Kindlin regulates Rho GTPases, including RhoA, Rac1, and Cdc42, by modulating the activity of their upstream regulators, such as GEFs andGAPs. These small GTPases play a crucial role in controlling cell morphology and motility, and kindlin orchestrates their activity primarily through its interactions with integrins. By binding to the cytoplasmic tails of integrins, kindlin promotes integrin activation and clustering at FAs, which subsequently recruit and organize signaling complexes that regulate Rho GTPase activity. For instance, kindlin enhances the activity of GEFs, facilitating the exchange of GDP to GTP on Rho GTPases, thereby activating them. This mechanism is particularly evident in kindlin-2′s regulation of Rac1 activity, which drives lamellipodia formation and accelerates cell migration. Concurrently, kindlin modulates the localization or activity of GAPs to ensure the appropriate inactivation of Rho GTPases, balancing cytoskeletal dynamics. Through these interactions, kindlin enables the spatial and temporal coordination of Rho GTPase activation, promoting lamellipodia at the leading edge via Rac1 and stabilizing stress fibers at the rear through RhoA [78]. Furthermore, kindlin serves as an adaptor protein at FAs, assembling complexes that include IIK and FAK, further influencing cytoskeletal reorganization. These regulatory activities underscore kindlin’s pivotal role in cell motility and structural stability [79].

Kindlin plays a pivotal role in regulating the dynamic assembly and dissolution of adhesion sites, processes essential for effective cell migration. During migration, the formation and breakdown of adhesion sites are tightly coordinated to allow the cell to extend its leading edge and retract its trailing edge. Kindlin interacts with FA proteins such as paxillin and talin to stabilize adhesion sites and mediate integrin activation, ensuring proper linkage between the ECM and the cytoskeleton. Through these interactions, kindlin modulates Rho GTPase activity, promoting Rac1-driven lamellipodia formation at the leading edge and RhoA-induced contractility at the trailing edge, thus orchestrating cell motility. Additionally, kindlin directly interacts with F-actin via its actin-binding K2 domain, reinforcing the cytoskeletal network and contributing to adhesion site stability and remodeling. These combined activities underscore kindlin’s critical role in the spatial and temporal regulation of adhesion dynamics and cytoskeletal organization during cell migration [17,80].

### 3.3. Abnormal Functions and Diseases of Kindlin

The mutation or dysfunction of kindlin is associated with various diseases. In addition to the previously mentioned KS and LAD, the aberrant expression of kindlin in tumors is a current research focus.

KS, associated with mutations in kindlin-1, is a rare autosomal recessive genetic disorder characterized by skin fragility, blistering, chronic ulcers, photosensitivity, and progressive poikiloderma—a combination of skin atrophy, pigmentation changes, and telangiectasia. Additionally, patients may develop complications such as periodontal disease and an increased risk of skin cancers due to persistent inflammation and defective barrier function [81,82]. The absence of functional kindlin-1 disrupts integrin-mediated adhesion and signaling in keratinocytes, leading to impaired cell adhesion and weakened structural integrity of the skin. The understanding of kindlin-1′s role in cell adhesion and cytoskeletal dynamics has provided insights into the pathogenesis of this syndrome and highlights its importance in maintaining epithelial tissue integrity [73].

Research indicates that kindlin-2 is frequently upregulated in various cancers, including breast, colorectal, and lung cancers, where it drives cancer cell migration and invasion [83]. For example, Wei et al. [84] demonstrated that elevated kindlin-2 levels facilitate tumor progression and angiogenesis in melanoma via the mammalian target of the rapamycin/vascular endothelial growth factor A signaling pathway. Similarly, Sossey-Alaoui et al. [85] reported that kindlin-2 is crucial for the EMT in breast cancer, linking its overexpression to aggressive tumor behavior. Furthermore, studies have shown that kindlin-2 enhances the invasiveness of gastric cancer cells by phosphorylating integrins, thereby facilitating cell adhesion and migration [86].

In addition to its involvement in solid tumors, kindlin-3 has also been linked to immune system regulation. Abnormal regulation of kindlin-3 can lead to immune deficiencies, as its absence is associated with LAD, which compromises the immune response and increases susceptibility to infections [87]. These findings underscore the dual functions of kindlin proteins in both cancer biology and immune function. Moreover, kindlin-2 expressions within the tumor microenvironment have been associated with macrophage infiltration, further influencing tumor progression. Sossey-Alaoui et al. demonstrated that kindlin-2 regulates tumor–stromal interactions by modulating the recruitment of macrophages, a key process in tumor growth and metastasis [88]. These findings suggest that kindlin-2 influences cancer cells directly while also altering the tumor microenvironment to favor malignancy.

## 4. The Mechanism of Interaction Between Paxillin and Kindlin

Paxillin and kindlin jointly mediate cell adhesion, migration, and integrin signaling. Beyond integrin activation, they coordinate ECM signals into intracellular responses. Their interaction is crucial for integrin function, and lacking either protein significantly disrupts cellular adhesion, migration, and signaling.

### 4.1. The Synergistic Role of Paxillin and Kindlin in Integrin Activation

Paxillin and kindlin interact with distinct regions of the integrin β-subunit to establish a crucial signaling network for integrin activation. This cooperation stabilizes integrins in their active conformation, promoting signaling pathways that regulate adhesion dynamics, cytoskeletal organization, and cell migration. For example, paxillin recruits kinases such as Src and FAK, while kindlin facilitates integrin clustering, ensuring efficient cell–ECM adhesion. Kindlin binds to the NPXY motif on the integrin’s intracellular tail through its FERM domain, switching integrins from a low- to high-affinity state. Talin supports this by binding the integrin tail, aiding kindlin in sustaining integrin activation [89,90,91]. This cooperative action is essential because the absence of either paxillin or kindlin leads to incomplete integrin activation, disrupting cell adhesion and downstream signaling [67,92,93].

In addition to its structural role, paxillin also actively recruits key kinases such as Src and FAK to FAs, which are necessary for propagating downstream signals after integrin activation [94]. This recruitment enhances integrin activation efficiency and ensures the effective transmission of signals required for cellular adhesion and migration. Notably, paxillin and kindlin jointly regulate conformational changes in integrins during the early stages of activation, which strengthen cell adhesion to the ECM [90]. Kindlin enhances integrin affinity, thereby increasing adhesion strength, whereas paxillin ensures the effective propagation of downstream signaling events [63,95].

### 4.2. Paxillin and Kindlin in the Regulation of Focal Adhesion Dynamics and Cell Migration

FAs are flexible complexes that mediate cell–ECM interactions via integrins, enabling the regulation of cellular adhesion, migration, and cytoskeletal dynamics. Paxillin and kindlin are key regulators in both FA formation and disassembly, facilitating cellular adaptation to mechanical conditions.

The assembly and disassembly of FAs are tightly regulated processes involving coordinated interactions among proteins such as kindlin, talin, paxillin, Src, and FAK. FA assembly begins with talin and kindlin-mediated integrin activation, which transitions integrins from a low-affinity to a high-affinity state [90]. Kindlin binds to the β-integrin cytoplasmic tail, facilitating integrin clustering and initiating the recruitment of talin, which links integrins to the actin cytoskeleton and reinforces the integrin–ECM connection. Talin also recruits vinculin, further stabilizing nascent FAs. Paxillin contributes to FA maturation by binding integrins and acting as a scaffold for signaling molecules. Through its LD motifs, paxillin interacts with kinases like FAK and Src, driving their activation and triggering phosphorylation events that strengthen the adhesion complex. These events recruit additional proteins, promote cytoskeletal remodeling, and enhance FA resistance to mechanical forces, enabling stable cell adhesion and motility [29,96,97].

A disassembly is equally critical and involves integrin deactivation and protein detachment. Kindlin facilitates the release of integrins from their high-affinity state, allowing detachment from the ECM. Concurrently, paxillin regulates disassembly by interacting with FAK and Src, which activate signaling cascades to recruit proteases like calpain, cleaving adhesion proteins and breaking down the FA structure. The phosphorylation state of paxillin plays a pivotal role in this process, altering its binding affinities and shifting its function from adhesion stabilization to disassembly. For instance, specific phosphorylation events recruit molecules that deactivate integrin-binding proteins or promote cytoskeletal contraction, aiding in cell detachment [15,98,99]. This coordinated interplay between kindlin, paxillin, and associated kinases ensures precise FA turnover, enabling efficient cell migration and adaptability to mechanical forces. However, further research is needed to fully elucidate the temporal and spatial regulation of these interactions [29,100].

### 4.3. Coordinated Regulation of Cell Migration by Paxillin and Kindlin

Paxillin and kindlin work together to regulate the dynamic balance between FA at the leading edge of the cell and its disassembly at the rear edge during migration. Kindlin stabilizes integrins at the leading edge, ensuring robust ECM adhesion, which is essential for forward cell movement [80,101,102]. Simultaneously, paxillin orchestrates FA disassembly at the trailing edge by interacting with key kinases and adhesion proteins, facilitating cell detachment. This coordinated activity ensures that the cell remains anchored at the front while releasing adhesion at the rear, allowing efficient movement [36,103,104].

In addition to adhesion regulation, paxillin and kindlin jointly modulate cytoskeletal reorganization, a process critical for cell migration. By activating integrins, these proteins influence the activity of Rho GTPases, such as RhoA, Rac1, and Cdc42, which are central regulators of the cytoskeleton. Paxillin promotes RhoA activation, inducing the formation of stress fibers that maintain cell tension, whereas Rac1 and Cdc42 contribute to the extension of membrane protrusions such as ruffles and lamellipodia, which are critical for cell motility [105,106]. Kindlin enhances these localized signaling effects by anchoring integrins to the ECM, ensuring that cytoskeletal changes are synchronized with FA dynamics.

## 5. Paxillin and Kindlin in Signal Transduction

Paxillin and kindlin are key regulators in integrin signaling, involved in pathways that support cell adhesion, migration, and cytoskeletal reorganization through interactions with cytoskeletal proteins, kinases, and GTPases. These molecules not only contribute to FA structure but also control cell proliferation, survival, and differentiation by connecting multiple signaling networks. This section focuses on their roles in key pathways such as Rho GTPase, MAPK, and PI3K/Akt, and highlights their functional differences across cell types.

### 5.1. Rho GTPase Signaling Pathway

The Rho family GTPases (RhoA, Rac1, and Cdc42) are essential for cytoskeletal reorganization, cell migration, and polarity. Paxillin and kindlin control cell shape and movement through the integrin-mediated activation of Rho GTPases. During cell migration, paxillin regulates directional movement by coordinating signaling pathways that control the cytoskeleton and cell adhesion. Through its LD4 motif, paxillin binds to G protein-coupled receptor kinase-interacting protein 1 (GIT1), which acts as a scaffold for signaling complexes. This interaction enhances the recruitment and activation of ADP-ribosylation factor-GTPase-activating proteins (ArfGAPs), which regulate Arf GTPases. GIT1, in turn, modulates Rho GTPase activity, particularly Rac1 and RhoA, to maintain cell polarity and motility. At the leading edge of migrating cells, GIT1 promotes Rac1 activation, driving lamellipodia formation through actin polymerization and FA stabilization. At the trailing edge, GIT1 suppresses RhoA activity, facilitating adhesion disassembly and tail retraction. This coordinated regulation balances protrusive and contractile forces, ensuring forward motion. Additionally, ArfGAPs deactivate Arf GTPases by accelerating GTP hydrolysis, enabling vesicle trafficking to deliver integrins and other adhesion molecules to the leading edge for new adhesion site formation. Simultaneously, ArfGAPs promote the endocytosis of integrins at the trailing edge, supporting adhesion disassembly and recycling. Together, paxillin’s interaction with GIT1 and the recruitment of ArfGAPs establish a signaling hub that integrates Rho and Arf GTPase activity, orchestrating cytoskeletal remodeling and FA turnover for efficient directional migration [23,107]. Furthermore, paxillin facilitates adhesion disassembly by interacting with ArfGAP, a key step in regulating migration speed [56,108].

Kindlin indirectly modulates the activity of Rho GTPases during integrin-mediated adhesion site formation by interacting with talin and the intracellular tail of integrins. Kindlin activates RhoA through integrin-activated signaling pathways, which in turn regulate the formation and contraction of actin fibers [109,110]. Additionally, kindlin interacts with small GTPase regulatory proteins, such as Tiam1, influencing Rac1 activity and ultimately contributing to cell migration and cytoskeletal reorganization [111,112].

### 5.2. MAPK Signaling Pathway

The MAPK signaling pathway regulates cell proliferation, survival, differentiation, and migration. Paxillin and kindlin influence MAPK activation via FAK and Src kinases. Paxillin is a key substrate of FAK and Src. Upon integrin activation, the autophosphorylation of FAK enables it to recruit Src kinase, further activating the downstream MAPK pathway. Paxillin, through its phosphorylated form, recruits growth factor receptor-bound protein 2 (GRB2) and son of sevenless (SOS), which are responsible for activating Ras, thereby initiating the MAPK signaling cascade [113]. MAPK pathway activation enhances both cell proliferation and survival, and its excessive activation in tumor cells often leads to uncontrolled cell growth [114].

Kindlin indirectly regulates the MAPK signaling pathway by facilitating integrin activation and FA complex assembly, though the precise mechanisms remain unclear. Kindlin binds to the cytoplasmic tail of β-integrins and works synergistically with talin to stabilize integrins in their high-affinity state. This process enhances integrin clustering and adhesion to the ECM, which is essential for the formation of FA complexes. Through kindlin-mediated FA assembly, FAK and Src kinase are recruited and activated [115].

FAK undergoes autophosphorylation at specific tyrosine residues, creating docking sites for Src and other adaptor proteins. Together, FAK and Src phosphorylate downstream targets such as paxillin. Paxillin, as a key substrate of FAK and Src, is phosphorylated and serves as a docking platform for proteins like GRB2 and SOS, which are responsible for activating Ras, a small GTPase that initiates the MAPK signaling cascade. Kindlin indirectly ensures efficient MAPK signal propagation by modulating integrin activation and FA stability. Specifically, the activation of Ras triggers the sequential activation of RAF, MEK, and ERK, amplifying the signals that regulate essential cellular processes such as proliferation, differentiation, and survival. Thus, kindlin acts as a critical upstream regulator connecting ECM interactions to intracellular MAPK signaling. However, the specific molecular links between kindlin and the MAPK pathway require further investigation to fully elucidate its precise role in signaling transduction [116].

### 5.3. PI3K/Akt Signaling Pathway

Paxillin promotes PI3K activation by acting as a scaffold for FAK and Src. Upon integrin activation, FAK autophosphorylates and recruits Src, forming a signaling complex. Paxillin is then phosphorylated, creating docking sites for PI3K. This activation leads to the production of phosphatidylinositol 3,4,5-trisphosphate (PIP3), which recruits and activates Akt. Akt subsequently triggers downstream signals that regulate cell survival, proliferation, and metabolism. Through its interactions with FAK and Src, paxillin links integrin activation to the Akt pathway. As a central pathway for survival and proliferation, the activation of PI3K/Akt typically enhances cells’ anti-apoptotic capacity, as well as their migratory and adhesive abilities [117]. Paxillin’s role in this pathway highlights its significance in both physiological and pathological conditions, especially in tumorigenesis. At adhesion sites, phosphorylated paxillin can directly or indirectly stimulate PI3K activation, leading to subsequent Akt activation. The dysregulated activation of the PI3K/Akt pathway by paxillin is frequently associated with enhanced invasiveness in tumor cells [118].

Kindlin indirectly supports the PI3K/Akt pathway by stabilizing the integrin–FAK complex and facilitating the assembly of signaling molecules essential for the activation of downstream pathways. Kindlin binds to the cytoplasmic tail of β-integrins, working in synergy with talin to stabilize integrins in their active, high-affinity conformation. This stabilization promotes integrin clustering and firm adhesion to ECM, a critical step in the formation of FAs. Upon integrin activation, kindlin contributes to the recruitment and activation of FAK, which undergoes autophosphorylation at tyrosine 397. This phosphorylation creates a docking site for Src kinase, leading to the formation of a FAK-Src complex. The complex further phosphorylates downstream targeting molecules, including PI3K, which is recruited to FAs through adaptor proteins.

Activated PI3K converts phosphatidylinositol 4,5-bisphosphate (PIP2) into PIP3, a critical lipid second messenger that recruits Akt to the plasma membrane via its PH domain. Akt is then phosphorylated and activated by upstream kinases such as PDK1 and mTORC2. Once activated, Akt triggers a range of downstream signals that promote cell survival, growth, proliferation, and resistance to apoptosis. In contexts where kindlin is highly expressed, such as in tumor cells, its role in stabilizing the integrin–FAK complex and promoting efficient signaling propagation becomes even more pronounced. High kindlin expression enhances the ability of tumor cells to adhere to the ECM, resist detachment-induced cell death (anoikis), and survive under unfavorable conditions. This facilitates tumor cell proliferation, migration, and metastasis by ensuring robust signaling through the PI3K/Akt pathway. Through these mechanisms, kindlin acts as a crucial mediator that links integrin-mediated ECM interactions to intracellular survival and growth signals [115,116].

## 6. Paxillin and Kindlin in Disease

**Figure 2:**
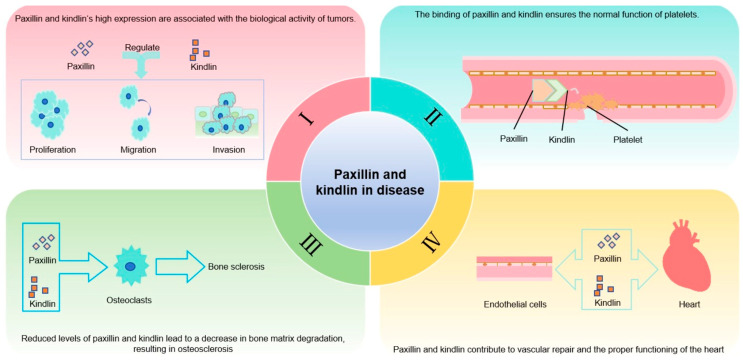
Paxillin and kindlin in various diseases. (**I**) The high expression of paxillin and kindlin promotes tumor cell activity. (**II**) The interaction between paxillin and kindlin ensures the proper functioning of platelets. (**III**) The disruption of kindlin or paxillin in osteoclasts leads to osteosclerosis. (**IV**) Paxillin and kindlin also contribute to vascular endothelial and cardiac functions.

The contributions of paxillin and kindlin are also evident in the physiology of various diseases. Figure 2 illustrates some of the currently identified aspects of their pathophysiological synergy.

### 6.1. Tumor Progression

#### 6.1.1. Regulatory Roles in the Tumor Microenvironment

In the tumor microenvironment, paxillin and kindlin play pivotal roles by interacting with integrins to regulate cellular responses to the ECM. These interactions drive processes such as tumor cell proliferation, migration, and angiogenesis. For instance, paxillin enhances endothelial cell adhesion and expansion during angiogenesis, while kindlin-2 promotes cancer cell invasion through integrin β1 activation [88,119,120].

Paxillin modulates angiogenesis by enhancing integrin signaling; when angiogenic factors like VEGF are released, paxillin activates integrins, promoting endothelial cell adhesion and expansion, thereby increasing angiogenic capacity [58]. Similarly, kindlin-2 supports tumor angiogenesis by promoting vascular endothelial cell migration and proliferation through integrin β1 activation, essential for tumor vasculature formation [76,119,121].

#### 6.1.2. High Expression in Invasive Tumors

Elevated expression levels of paxillin and kindlin-2 are recognized as important biomarkers associated with the aggressive behavior and progression of various invasive cancers. The upregulation of these genes significantly increases the migratory, invasive, and survival properties of cancer cells by influencing critical signaling pathways, such as the PI3K/Akt and MAPK pathways, ultimately leading to worse clinical outcomes in affected patients. Research has particularly demonstrated a strong link between high paxillin expression and more aggressive forms of cancers, including those of the breast, colon, and lung, with this overexpression tied to a poorer prognosis. Increased paxillin levels enhance tumor cell proliferation, migration, and invasion by activating key signaling cascades, notably the PI3K/Akt and MAPK pathways [122]. The phosphorylation of paxillin is crucial for enabling tumor cell migration and invasion, highlighting its role as an oncogenic factor in various malignancies [54,123].

Similarly, kindlin-2 has been widely researched for its involvement in invasive cancers. The overexpression of kindlin-2 has been documented in pancreatic and prostate cancers, where it is directly associated with enhanced invasiveness and the metastatic potential of cancer cells [124,125]. Kindlin-2 enhances integrin β1 activation, which subsequently accelerates cancer cell invasion and metastasis via downstream signaling pathways such as FAK and Src [119,126]. Furthermore, the interaction of kindlin-2 with paxillin is critical for the formation of FAs, which are necessary for cell migration and invasion. This interaction not only stabilizes adhesion sites but also integrates various signaling pathways that facilitate tumor progression [90,127].

### 6.2. Nontumor Diseases

#### 6.2.1. Platelet Dysfunction

Kindlin-3 is crucial for the activation of integrin αIIbβ3 in platelets, with its interaction with talin and paxillin playing an essential role in maintaining normal platelet function [92,113]. Researchers have demonstrated that kindlin-3 binding to paxillin ensures proper platelet adhesion and spreading [5]. Kindlin-3 deficiency disrupts platelet function, impairing blood coagulation and raising the bleeding risk, underscoring the importance of kindlin-3 and paxillin in platelet regulation.

#### 6.2.2. Osteosclerosis

Paxillin and kindlin are also involved in the process of bone remodeling. Klapproth et al. demonstrated that low levels of kindlin-3 expression lead to reduced osteoclast adhesion capacity, weakening the efficiency of bone matrix degradation and ultimately causing osteosclerosis [128]. Specifically, reduced expression of kindlin-3 leads to decreased phosphorylation at the Y31 and Y118 sites, thereby impairing the formation of functional sealing zones in osteoclasts and preventing efficient bone matrix degradation. Although partial adhesion structures can still form under conditions of low kindlin-3 expression, these structures are functionally insufficient, compromising the normal bone remodeling ability of osteoclasts [92,128]. Paxillin enhances the bone resorption capacity of osteoclasts through its interaction with actin and myosin IIA [129].

#### 6.2.3. Cardiovascular Diseases

Paxillin and kindlin play pivotal roles in the cardiovascular system by modulating key cellular processes such as migration, proliferation, and adhesion, which are essential for vascular repair, angiogenesis, and cardiac function. Paxillin supports vascular endothelial cell migration and proliferation through its interactions with FAK and Src. Upon integrin activation, FAK is recruited to FAs and autophosphorylated, creating a binding site for Src kinase. This FAK–Src complex phosphorylates paxillin, which serves as a scaffold for recruiting additional signaling molecules such as Grb2 and SOS, activating Ras and the downstream MAPK pathway. This signaling cascade promotes endothelial cell proliferation and migration, facilitating the formation of new blood vessels and the repair of damaged vasculature. Additionally, paxillin interacts with cytoskeletal components, coordinating actin dynamics that are essential for the directional migration of endothelial cells [130].

Similarly, kindlin-2 is crucial for maintaining cardiomyocyte function and structural integrity. Kindlin-2 binds to the cytoplasmic tails of β-integrins, promoting their activation and stabilizing integrin-mediated adhesion to ECM. This adhesion is essential for the mechanical stability of cardiomyocytes, which are subjected to continuous mechanical stress during cardiac contraction and relaxation. Kindlin-2 also regulates FA signaling by facilitating the recruitment of FAK and other signaling molecules, linking ECM interactions to intracellular pathways that maintain cardiomyocyte survival and function. Mutations or aberrant expression of kindlin-2 disrupt integrin activation and FA stability, leading to impaired signaling, cardiomyocyte dysfunction, and the progression of cardiovascular diseases such as dilated cardiomyopathy [131,132].

## 7. Paxillin and Kindlin as Potential Therapeutic Targets

Research on how paxillin and kindlin influence cell adhesion, migration, and signal transduction has identified these proteins as potential therapeutic targets, particularly for cancer treatment. Both proteins play crucial roles in cytoskeletal reorganization, integrin activation, and FA dynamics, supporting malignant behaviors in cancer, such as invasion, metastasis, and drug resistance. Targeted therapies for paxillin and kindlin focus on inhibiting or reversing these functions to mitigate cancer progression and improve patient outcomes.

### 7.1. Therapeutic Strategies Targeting Paxillin

Targeting paxillin and kindlin directly presents exciting opportunities but also significant challenges. Potential approaches include the use of peptidomimetics, nanobodies, and PROTACs (proteolysis-targeting chimeras). Peptidomimetics can mimic the structure of natural protein interfaces, blocking critical interactions such as those between paxillin and its binding partners (e.g., FAK, Src, and vinculin). These molecules can disrupt the signaling complexes required for cell adhesion, migration, and invasion. Nanobodies, which are small, stable antibody fragments, offer high specificity and can target unique epitopes on paxillin or kindlin. These could be used to block interactions or modulate protein function in cancer cells selectively. PROTACs, on the other hand, induce the selective degradation of paxillin or kindlin by recruiting them to E3 ubiquitin ligases, leading to proteasomal degradation. This approach could be particularly effective in cancers where paxillin or kindlin is overexpressed, reducing their abundance rather than merely inhibiting their function [33,133,134].

Disrupting protein–protein interactions (PPIs) with small molecules remains a significant challenge due to the nature of the interaction surfaces. Unlike enzyme active sites, which are typically small and well defined, PPIs involve large, flat, and dynamic surfaces that lack deep pockets for small molecules to bind effectively. This makes it difficult to achieve the high affinity and specificity needed for therapeutic targeting. Moreover, targeting proteins like paxillin and kindlin is complicated by their involvement in multiple signaling pathways, raising concerns about off-target effects and toxicity [135,136,137].

The isoform problem adds another layer of complexity, particularly with kindlin. Kindlin-2 is often overexpressed in cancer cells and plays a crucial role in promoting cancer cell survival, migration, and metastasis. It is a promising therapeutic target, but kindlin-3, another isoform, is essential for integrin activation in hematopoietic cells, including platelets and immune cells. Blocking kindlin-3 could result in severe side effects such as impaired blood clotting and immune dysfunction. This highlights the need for isoform-specific targeting strategies, which could be achieved by designing molecules that selectively bind to regions unique to kindlin-2 or by developing isoform-specific PROTACs or nanobodies [138,139].

### 7.2. Therapeutic Strategies Targeting Kindlin

Kindlin, similar to paxillin, is pivotal in integrin activation, cell migration, and metastasis, with distinct isoforms (kindlin-1, kindlin-2, and kindlin-3) contributing specific roles in various tissues. Targeting kindlin’s interaction with integrins through small-molecule inhibitors has been shown to curb cancer cell adhesion and invasion, especially in cases where kindlin-2 is overexpressed [86,140]. Beyond cancer, kindlin-3 is essential for immune cell adhesion, presenting therapeutic potential for immune-related disorders like LAD III, further indicating its role in immunotherapies [141,142]. Additionally, kindlin serves as a tumor biomarker, with its upregulation in cancers such as pancreatic and breast cancer correlating with poor prognosis and aiding in survival and treatment predictions [88,119,143].

### 7.3. Combination Therapy Targeting Paxillin and Kindlin

Due to their collaborative roles in adhesion, migration, and signaling, targeting both paxillin and kindlin offers a promising antitumor strategy. Small-molecule inhibitors disrupting paxillin–FAK interactions and kindlin–integrin binding have shown potential in reducing cancer invasiveness. Furthermore, advanced techniques like CRISPR/Cas9 could be employed to selectively inhibit their expression. However, challenges remain, including potential off-target effects and the need for precise delivery systems [144,145].

## 8. Future Research Directions

Although significant progress has been made in elucidating the roles of paxillin and kindlin in cell adhesion, signaling, and disease mechanisms, many questions remain unanswered. Future research should focus on their involvement in broader processes such as stem cell differentiation, apoptosis, and tissue regeneration. Additionally, further exploration of their therapeutic potential, particularly in cancer and immune disorders, could open new avenues for targeted treatments.

### 8.1. Paxillin and Kindlin in Novel Cellular Processes

TPaxillin and kindlin are integral not only to integrin-regulated cell adhesion, migration, and signal transduction but also to broader cellular processes like proliferation, differentiation, and apoptosis, which remain underexplored. Emerging evidence suggests that paxillin and kindlin influence these processes by interacting with non-integrin signaling pathways such as Hippo, Wnt, and PI3K/Akt. In the context of cell proliferation and differentiation, paxillin, through its role as a scaffold protein, may recruit signaling molecules that regulate Hippo pathway activity, particularly YAP/TAZ. YAP/TAZ are key transcriptional regulators of genes that control stem cell renewal and differentiation, and paxillin’s influence on their localization and activity could prove vital for stem cell biology. Similarly, kindlin, via its integrin-binding capacity, stabilizes cell–ECM adhesions essential for maintaining stem cell niches. Kindlin’s role in controlling cell polarity and integrin clustering suggests it could regulate differentiation cues in various tissue microenvironments.

For apoptosis and survival, paxillin is a known modulator of tumor cell survival, particularly through the PI3K/Akt and ERK/MAPK pathways. By serving as a docking site for upstream activators like FAK and Src, paxillin facilitates Akt phosphorylation, which inhibits pro-apoptotic proteins such as Bad. Kindlin’s potential involvement in apoptosis may be linked to its interaction with paxillin and its ability to influence integrin-mediated signaling. Understanding how these proteins jointly regulate apoptosis could provide new insights into tumor cell resistance to apoptosis and highlight therapeutic strategies targeting these pathways.

### 8.2. Paxillin and Kindlin in Tissue Repair

In tissue repair, paxillin orchestrates cytoskeletal dynamics and FA turnover, enabling keratinocytes and fibroblasts to migrate into the wound site. Its interactions with FAK and Src coordinate the assembly of FA complexes and downstream signaling cascades, such as MAPK, that drive cell proliferation and migration. Kindlin complements this role by stabilizing integrin activation and clustering, enhancing ECM adhesion and ensuring FA stability under mechanical stress. The interplay between paxillin and kindlin at FA sites regulates the integration of chemical and mechanical signals, crucial for wound closure and tissue remodeling.

In regenerative medicine, the paxillin–kindlin axis could be exploited to optimize scaffold–cell interactions in tissue engineering. Kindlin’s role in integrin activation ensures robust cell adhesion to biomaterial scaffolds, while paxillin’s ability to regulate cytoskeletal organization enhances cell spreading, migration, and proliferation. Future studies need to explore how biomaterial properties, such as stiffness and topography, influence paxillin and kindlin activity to accelerate tissue regeneration.

### 8.3. Paxillin and Kindlin Interactions with Other Signaling Pathways

Paxillin and kindlin’s influence also extends to pathways like Hippo and Wnt. Paxillin interacts with YAP/TAZ, effectors of the Hippo pathway, through its role in FA turnover and cytoskeletal dynamics. By modulating the mechanical signals transmitted through FAs, paxillin may regulate YAP/TAZ nuclear translocation and transcriptional activity, impacting cell proliferation and apoptosis. Similarly, kindlin’s role in mechanotransduction could link cytoskeletal tension to Hippo signaling, influencing tissue architecture and cell survival under mechanical stress. The functional interplay between kindlin and YAP/TAZ represents a promising avenue for research, particularly in contexts where tissue homeostasis is disrupted, such as cancer or fibrosis [146].

In the Wnt/β-catenin pathway, kindlin is known to regulate β-catenin stabilization and transcriptional activity via integrin activation. Paxillin’s role in this pathway, while less understood, may involve its interaction with Src and other kinases that influence Wnt signaling. Investigating paxillin’s potential regulation of β-catenin could provide insights into its role in cell fate determination and tumor progression. Understanding these mechanisms in detail is crucial, as they provide a foundation for therapeutic interventions targeting paxillin and kindlin in diseases like cancer and tissue degeneration.

### 8.4. Future Challenges and Opportunities in Research

Despite extensive research on paxillin and kindlin, several challenges remain. The multifunctional roles and cell-specific diversity of these proteins complicate the understanding of their full functions. Moreover, most studies focus on vitro models; future research should prioritize in vivo validation, especially in mouse models and clinical applications.

Emerging technologies like single-cell RNA sequencing, super resolution microscopy, proteomics, and CRISPR/Cas9 gene editing offer precise tools to explore the molecular networks controlled by paxillin and kindlin. These advancements are set to deepen insights into their roles in health and disease, informing new therapeutic strategies. Future research can harness these technologies to clarify paxillin and kindlin networks and develop targeted, personalized therapies.

## 9. Conclusions

Paxillin and kindlin are essential regulators of cell adhesion, signaling, and tumor progression. While substantial progress has been made in understanding their functions, numerous aspects of their roles in cellular physiology and disease remain to be explored. Future research is expected to uncover novel mechanisms involving these proteins, paving the way for innovative therapeutic strategies in cancer, immune disorders, and regenerative medicine.

## Data Availability

No new data were created or analyzed in this study. Data sharing is not applicable to this article

## Abbreviations

Akt	Activation of _rotein Kinase B
ArfGAP	ADP-Ribosylation Factor-GTPase-Activating Protein
ECM	Extracellular Matrix
EMT	Epithelial-to-Mesenchymal Transition
ERK	Extracellular Signal-Regulated Kinase
FA	Focal Adhesion
FAK	Focal Adhesion Kinase
GAP	GTPase-Activating Protein
GEF	Guanine Nucleotide Exchange Factor
GIT1	G Protein-Coupled Receptor Kinase-interacting Protein 1
GRB2	Growth Factor Receptor-Bound Protein 2
IIK	Integrin-Linked Kinase
KS	Kindler Syndrome
LAD	Leukocyte Adhesion Deficiency
LAD III	Leukocyte Adhesion Deficiency type III
MAPK	Mitogen-Activated Protein Kinase
PH	Pleckstrin Homology
PI3K	Phosphoinositide-3-Kinase
PIP2	Phosphatidylinositol 4,5-bisphosphate
PKC	Protein Kinase C
PPI	Protein–Protein Interaction
PtdIn	Phosphatidylinositol
SFKs	Src family Kinases
SOS	Son Of Sevenless
